# Blockchain-Based Federated Learning System: A Survey on Design Choices

**DOI:** 10.3390/s23125658

**Published:** 2023-06-16

**Authors:** Yustus Eko Oktian, Sang-Gon Lee

**Affiliations:** College of Software Convergence, Dongseo University, Busan 47011, Republic of Korea

**Keywords:** blockchain, smart contract, federated learning, design pattern

## Abstract

The vanilla federated learning is made for a trusted environment, while in contrast, its actual use cases require collaborations in an untrusted setting. For this reason, using blockchain as a trusted platform to run federated learning algorithms has gained traction lately and has become a significant research interest. This paper performs a literature survey on state-of-the-art blockchain-based federated learning systems and analyzes several design patterns researchers often take to solve existing issues through blockchain. We find about 31 design item variations throughout the whole system. Each design is further analyzed to find pros and cons, considering fundamental metrics such as robustness, efficiency, privacy, and fairness. The result shows a linear relationship between fairness and robustness in which, if we focus on improving fairness, it will indirectly become more robust. Furthermore, improving all those metrics altogether is not viable because of the efficiency trade-off. Finally, we classify the surveyed papers to spot which designs are popular among researchers and determine which areas require immediate improvements. Our investigation shows that future blockchain-based federated learning systems require more effort regarding model compression, asynchronous aggregation, system efficiency evaluation, and the application for cross-device settings.

## 1. Introduction

Federated Learning (FL) [1] has gained traction recently due to its ability to train neural network models on local machines and protect user data privacy. This popularity comes from the increased interest in guarding data privacy (e.g., through the GDPR law [2]) and the fact that FL is advertised by big tech giants such as Google [3]. Unfortunately, vanilla FL [1] opens yet another novel security, privacy, and trust issue previously unavailable in the old platform. For starters, FL requires multiple participants to contribute to training a single global model. The high frequency of model sharing makes it easier for adversaries (disguised as valid clients) to obtain trained models for free. Malicious participants can also perform low-effort training to corrupt the global model. Furthermore, Carlini et al. [4] demonstrate the membership-inference attack, which allows attackers to guess the trained private data from a given trained model. This attack allows adversaries to obtain private data in the FL setting. Finally, participants will not trust one another, and it will be hard to persuade them to cooperate honestly. The vanilla FL works well because they assume the system is run in a trusted environment, where a single company owns all the infrastructure. However, the true potential of FL use cases is in a distributed environment, where many participants can collaborate to choose which training jobs they want and obtain a fair incentive upon successfully training the global model. Hence, the trust issue becomes a significant problem in realizing that goal.

Recent advances in blockchain have revolutionalized existing architectures to lean more towards decentralization. Bitcoin [5], in particular, has proven itself to allow various untrusted parties to distribute or exchange money in a secure, trusted, and decentralized manner without the intervention of a single authority. Other blockchain platforms such as Ethereum [6] even allow users to run code in the blockchain network through a smart contract. The code execution within the blockchain can be performed securely and trusted, which opens many new opportunities (such as distributed FL) to be run in a trusted manner. For this reason, integrating blockchain into FL will bring tremendous benefits. Blockchain provides hard-to-tamper and non-repudiation guarantees for the stored data in its ledger. Hence, storing local/global models (or their metadata) in blockchain can help improve the overall integrity of the FL system. Blockchain nodes also maintain the stored data collaboratively. Together, they can audit the stored model/metadata and detect any malicious intents from trainers. Finally, the smart contract execution is also deterministic, which makes the smart contract become the new root of trust for all trainers to believe that the FL process (e.g., training, review, aggregation) has been performed correctly and fairly.

Our argument on naming blockchain as a suitable candidate for FL is backed up by the fact that many researchers in the literature are also integrating blockchain into their FL system [7,8]. However, because no known standard exists, those researchers often take customized approaches. This situation creates a lot of system-design variations and stresses the necessity to find the design patterns and study them in detail. Analyzing trends in commonly used approaches and pointing out which areas lack or need improvement also become important. Such investigation can be beneficial to drive future research on blockchain-based FL systems on the right path, which becomes our motivation to propose this survey.

### 1.1. Contributions

This paper examines the state-of-the-art blockchain-based FL systems to analyze their progress, trends, and possible future research directions. We study the surveyed papers in detail to extract and analyze specific system-design parameters. In summary, we made the following contributions:

- We investigate research papers from the literature and find about 31 design variations researchers often take to solve FL issues in their system.
- We analyze the pros and cons of committing to each design pattern concerning the fundamental FL metrics such as robustness, efficiency, privacy, and fairness.
- We classify the surveyed papers to seek trends of popular design patterns.
- Based on our analysis, we derive several research directions to enhance the quality of future blockchain-based FL systems.

### 1.2. Related Surveys

Several surveys related to the FL in general [9] or blockchain-based FL systems such as [7,8] exist in the literature. However, to the best of our knowledge, survey papers that discuss detailed design patterns of blockchain-based FL systems, analyze their pros and cons, and derive future directions based on the investigated design trends are not yet available.

### 1.3. Paper Organization

The rest of this paper is organized as follows. Section 2 provides an overview of the FL system and the motivation behind why blockchain is needed for FL. Section 3 introduces our findings on blockchain-based FL system design patterns. We then analyze the pros and cons of each found design pattern in Section 4. We discuss classifications of surveyed papers, future research directions, and limitations of our survey in Section 5. Finally, we conclude in Section 6.

## 2. Blockchain Integration into Federated Learning

### 2.1. Brief Overview of Federated Learning System

**Figure 1:**
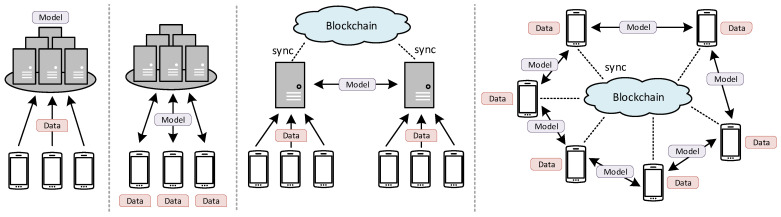
A comparison between various machine-learning architectures. From left to right: centralized ML, vanilla FL, blockchain-based cross-silo FL, blockchain-based cross-device FL.

Vanilla FL aims to train a global model collaboratively in devices’ machines. Instead of sending private data to a server for remote training (c.f., the leftmost image from Figure 1), the server delivers the model to devices for local training (c.f., the second left from Figure 1). After training, devices send the trained model back to the server for aggregation. By employing this strategy, the private data never leave the devices, and therefore, we can protect user data privacy.

The vanilla FL only considers devices belonging to one organization. However, its use cases can be extended to support multiple organizations. Since now multiple untrusted entities are involved in the FL process, blockchain can be used to synchronize the data and logic such that they can collaborate in a secure and trusted manner. Depending on the client’s data, FL processes can further be divided into cross-silo [10] or cross-device [11].

In a cross-silo setting, we perform data training on clusters of private servers belonging to a group of organizations within a consortium. Devices and servers in the same organization trust each other; therefore, private data can be sent safely from devices to servers for remote training. However, organizations do want to share data beyond their own infrastructure. In this case, they only share trained models with others and use FL to collaboratively train the data (c.f., second right from Figure 1). The cross-silo FL typically involves a small number of trainers (i.e., servers), and each holds a huge amount of training data.

On the other hand, devices directly train their data locally and share models with other devices without intermediary servers in the cross-device setting (c.f., the rightmost image of Figure 1). The sharing boundary is more restricted in this setting since devices do not trust each other and do not want to share private data beyond their machines. Furthermore, each device most likely has a small number of training data. Hence, we must recruit many devices to cope with this limited data source.

Finally, a typical FL platform has several roles (e.g., organizers, trainers, reviewers, and aggregators), and a given entity may play multiple roles simultaneously. The *organizers* act as initiators who set up the task by providing the necessary global model and training policy; they are also known as *model owners* in other studies. The *trainers* train the global model from the organizers. The *reviewers* evaluate the trained models from trainers. The *aggregators* collect the trained and evaluated local model parameters and combine them using specific aggregation algorithms. We will use these roles to describe FL entities throughout the rest of this paper.

### 2.2. Privacy, Security, and Trust Issues in Federated Learning System

While FL guards user private data by design, the vanilla FL system also opens novel privacy, security, and trust issues as consequences. Table 1 summarizes our discussion.

*Leakage*: The trained models become more susceptible to theft in FL than in conventional machine learning (ML) architecture. FL design encourages model sharing among entities, which generates a high probability of model leakage. Adversaries can quickly obtain a trained global model by spoofing or acting as valid clients. They do not need to compromise the server to get the model, as is usually required in ML. Moreover, even though attackers cannot obtain the private data, they can guess the trained data using a membership inference attack [4]. Therefore, private data leakage is still possible in the FL system.

*Poisoning*: Adversaries can perform two poisoning attacks in the FL system: (i) data poisoning and (ii) model poisoning attacks. The former allows attackers (as FL clients) to train their local model with lossy or low-quality data to disrupt the overall accuracy of the global model [12]. Meanwhile, attackers use adversarial examples in the local models to make the global model misclassify in the latter scenarios [13]. These two poisoning attacks are not unique to FL because they also exist in ML. However, those attacks are more accessible to be performed in FL than in ML due to the collaborative training that allows attackers to input malicious models by design. In contrast, the training is performed in a closed and secure environment in ML, which makes it difficult for adversaries to perform these attacks.

*Malicious Server*: Adversaries can hack the FL server and make it hostile. For example, a malicious server can intentionally choose only a few local updates from the clients (e.g., censoring or favoring a few clients), making it unfair to other honest clients. Attackers can also make the server malfunction, disrupting the entire FL process.

*Low Motivation and Dropouts*: Clients may not want to contribute to FL because of low motivation. From clients’ perspectives, the local training wastes their computing resources while only the server reaps the benefits from their processing power in the form of an accurate global model. From the server’s perspective, assuming that the clients are generous enough to train the global model, they may train it only for one, two, or a few epochs, then discontinue their process. The server will never get the desired result if many clients drop out during FL training. The cross-device FL setting suffers more damage from this issue because it requires more participants than the cross-silo one.

*Low Trust*: Participants do not trust each other by default, hindering collaboration to establish a resilient global model. FL system will lack adoption and usability without solving this issue because no one will join an untrusted environment.

### 2.3. Privacy, Security, and Trust Solutions for Federated Learning System

Looking at each FL issue independently, we find that existing research can solve those issues quite remarkably.

*Leakage*: We can apply several techniques to prevent data and model leakage. Differential privacy [14] can be used to protect private data against membership-inference attack [4]. The use of encryption [15] is very useful to guard the exchanged local and global models from unauthorized third-party. A registration procedure can also be made to limit access to the trained models from attackers [16]. Lastly, all model transfers among FL participants must be audited and logged accordingly for fast response leak detection [17].

*Poisoning*: A model evaluation is required to detect poisoning attacks in FL by analyzing each submitted local model. The aggregation server should have the policy to only accept local models with high accuracy. A local model trained with poisoned data (i.e., lossy or low-quality data) will most likely produce low accuracy during testing and, therefore, will be rejected. The server can also add adversarial example defense [18] to protect its global model against model poisoning attacks. Finally, a reputation system [19] can be added to punish malicious actors detected during the server evaluation process.

*Malicious Server*: The FL server poses security threats due to its centralized design. Hence, removing a single point of failure and making the system decentralized is an inevitable solution. We can borrow the technology from distributed systems such as Ethereum [6] or Hyperledger [20] to realize such decentralization.

*Low Motivation and Dropouts*: The incentive mechanism [21] can be applied to FL to encourage clients to train honestly with high-quality data. Meanwhile, to prevent clients from discontinuing the training arbitrarily, a deposit mechanism [22] can be made. We can boost the client’s trustability if we hold something valuable that the client owns, which, in this case, is the deposited funds.

### 2.4. Blockchain Roles in Federated Learning System

Despite our findings on solutions to the given FL issues, we still cannot fully solve the trust issues. Therefore, a trusted platform where the FL can be executed correctly is still highly required. This is where the blockchain can fit in by acting as the “missing” trusted platform that FL needs.

The trust value of blockchain comes from several reasons. First, sending transactions to the blockchain and smart contract requires the senders to sign their messages with digital signatures, providing auditable transaction records. Second, all data stored in the blockchain becomes hard to tamper because of the chain-of-hashes data structure and the consensus protocol. Third, the code execution in the smart contract is transparent and deterministic; hence, it is difficult to corrupt or hijack the smart contract.

Researchers often mix and match existing solutions with blockchain when building a blockchain-based FL system. In other words, a complete blockchain-based FL system can be considered a combination of many research ideas re-developed in a blockchain environment. For the Ethereum case, those solutions are rebuilt as smart contracts. These facts alone create many system design variations in the existing blockchain-based FL system, which we take as an opportunity to be surveyed and analyzed in this paper.

## 3. Design Patterns on Blockchain-Based FL System

**Figure 2:**
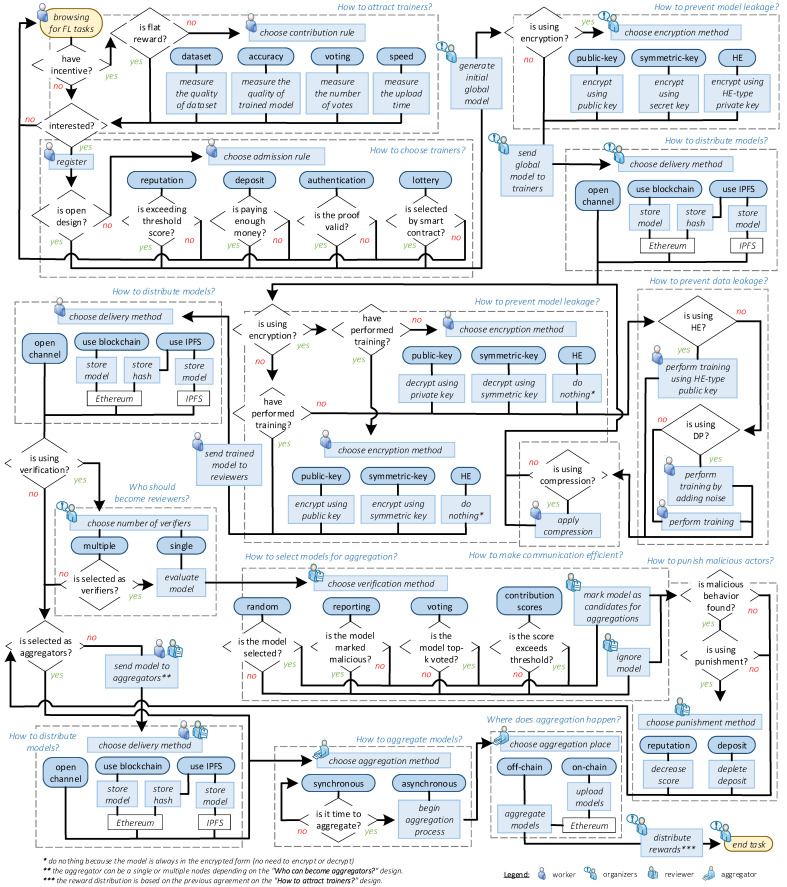
The workflow of a typical blockchain-based FL system (from registering clients to distributing, training, evaluating, and aggregating models) by considering design patterns from our survey.

A typical workflow of a blockchain-based federated learning system [23] can be divided into five stages: registration, distribution, training, evaluation, and aggregation. Our literature surveys outline several design patterns researchers often take at each stage. We extract those patterns and introduce them one by one in this section. Figure 2 summarizes our findings.

### 3.1. Survey Methodology

We gather research papers by using “*Federated Learning*”, “*Blockchain*”, “*Smart Contract*” and “*Ethereum*” as keywords from several publishing sites such as IEEE Explore, ScienceDirect, Web of Science, and Google Scholar. We filter those papers by title, abstract, sections, conclusion, figures, and tables. Afterward, we omit papers that do not relate to our “*Blockchain-Based Federated Learning*” theme. From these actions, we obtain 30 papers as candidates for our design-analysis survey.

Among those gathered materials, 43.33% comes from journals, 36.67% are obtained from conferences, 3.33% are thesis/dissertation, and 16.67% are pre-prints. Furthermore, we find a growing trend in the blockchain-based federated learning system in the literature, with 23.33% papers published before 2020 and 76.67% published in 2020 onward. Most contributors come from researchers with affiliations ties to Asia (58.62%), North America (17.24%), Europe (17.24%), and Australia (6.9%).

It is important to note that we only consider Ethereum [6] as our blockchain platform due to its usability and versatility. First, Ethereum is the second biggest cryptocurrency based on its Market Cap [24]. As a result, FL will most likely get adoptions on Ethereum rather than on custom blockchains as proposed in [25,26,27]. Ethereum is also flexible; if the poor performance of the current public Ethereum network hinders the FL applicability, then developers can build Ethereum on a private network [28], with minimal or no modifications needed in the smart contract code. Finally, the public Ethereum network has moved from Proof-of-Work (PoW) to Proof-of-Stake (PoS) consensus algorithm [29], which boosts its transaction-per second (TPS) while reducing the power consumption by magnitudes.

### 3.2. Registering FL Clients Strategy

Aside from generating initial global models, organizers must recruit some clients willing to train their models by employing registration mechanisms. The goal is to attract as many clients as possible to join the task and select which clients can become model trainers. This process is critical, especially in the cross-device setting.

#### 3.2.1. How to Attract Trainers?

Clients will join FL tasks only if they benefit from performing them. Therefore, the most efficient way to attract more clients to join is by giving them incentives. Our survey found two incentive strategies: using flat or contribution-based rewards.

**Flat Rewards**: Organizers treat all trainers equally by rewarding them with the same token reward after completing the FL task [30]. However, this method may not be fair to some trainers, especially those who can contribute more data or computing resources than others. Because all trainers will get the same reward, it may encourage them to train the models using the worst-effort instead of the best-effort approach. Hence, hurting the quality of the produced global models.

**Contribution-Based Rewards**: An alternative to boost fairness is to reward trainers differently based on their contributions to global models. The followings are several criteria to determine contributions.

*Dataset Quality*: We can assess the dataset quality based on two criteria: how much data is in the dataset and how unique the data is. Because vanilla FL [1] weighs the global model parameters based on the number of data used in training, organizers can reward clients that possess more data [31]. On the other hand, the uniqueness of data can be measured using other tools such as Earth Mover’s Distance (EMD) [32] or using Centroid Distance [33]. Organizers can then distribute more rewards to clients that can generate better scores in EMD [34] or Centroid Distance [33].

*Model Quality*: The data quality and computing capability affect the trained models’ accuracy. Local models trained with more data will most likely generate better accuracy than those trained with less data. Furthermore, local models trained with fewer local epochs (using lower computing resources) probably generate lower accuracy scores. Organizers can then give better rewards to trainers that can produce more accurate models [31].

*Voting*: Toyoda et al. [35] implement a voting mechanism on each submitted local model to determine top-k models. Based on the voting result, the system ranks the models and distributes the reward to trainers. The first-ranked model obtains the highest reward, while the k-th model obtains the lowest reward.

*Speed*: The rewards can also be distributed based on how quickly trainers complete the task. BlockFLA [36] measures how fast a particular trainer uploads the trained model to the system. Based on the recorded log, the earlier the clients submit, the greater the reward. In [34], the authors assess the trainers’ upload time based on the network bandwidth that the trainers have. The more bandwidth they have, the greater reward they can get.

#### 3.2.2. How to Choose Trainers?

After successfully attracting many clients to join, organizers must pick which clients can become trainers in their system.

**Open Trainer**: In open trainer design, clients may register as trainers in the system without any restriction or qualification required [37]. Organizers choose this open approach to allow many clients to join the system. With many registered clients, organizers can have a higher chance to include more data, resulting in a high-quality global model.

**Restricted Trainer**: Contrary to the former approach, clients must pass a qualification to be accepted as trainers in this latter method. The organizers may choose this strategy if they think that clients are untrusted. Several ways to restrict access to their systems can be described as follows.

*Reputation System*: One can use a reputation-based threshold such that only entities with more reputation scores than the required threshold can be accepted as workers [31].

*Deposit Mechanism*: Others may employ a fixed minimum deposit requirement to join the FL task, where any entity that can pay the deposit is eligible to become a worker, as in [38]. An exciting combination of reputation and deposit mechanism is employed in [31], such that entities with high reputation scores can join the system with a lower deposit amount than those with lower scores.

*User Authentication*: In some cases, organizers may choose only to approve specific trainers they trust. Those trainers are previously included in a list of trusted candidates. Afterward, organizers employ an authentication strategy, where entities must present secrets or proof (e.g., using the private key) that they belong to the trusted candidates. This pattern is used primarily on the cross-silo setting, e.g., in [39].

*Lottery*: Toyoda and Zhang [35] create a lottery system that randomly chooses eligible workers per training round from a pool of registered clients. Similarly, Zhang et al. [33] also selectively choose which clients can become workers depending on their current condition (e.g., whether physical devices are idle or charging). Fan et al. [34] propose an auction smart contract to determine the trainers’ eligibility. During client registration, clients must include their training capabilities, which can be determined from the training data size and the computing resources. Then, an auction algorithm in the smart contract elects trainer candidates based on the calculated capability scores. A candidate with a higher score will most likely be accepted as a trainer.

### 3.3. Distributing FL Models Strategy

After registration, organizers send the global model to trainers. Later, trainers will transmit the trained local models back to aggregators. The model delivery in FL is performed at the network level, separated from the application level, where we handle the training and aggregation. Therefore, it can be done in many ways.

#### 3.3.1. How to Distribute Models?

Three typical model-sharing strategies are used among surveyed papers.

**Through Open Channel**: The straightforward strategy is having entities create open Internet channels (e.g., REST API or web socket) that others can access. Entities then share their endpoints with the public by posting them to a well-known website or the blockchain. We can see this strategy is used in [33].

**Through Blockchain**: Since all surveyed papers leverage smart contracts and blockchain in their system, an alternative distribution approach is to use the deployed blockchain network as a medium for model transfer. In this scenario, the sender forms a transaction containing the shared models and then sends it to the smart contract, which will persist in the blockchain. The recipient, acting as a blockchain node, can query for the shared model in the blockchain once the underlying P2P blockchain consensus completes [36].

**Through IPFS**: The final alternatives propose storing models using a separate distributed storage system (such as InterPlanetary File System (IPFS) [40]) instead of using blockchain network [37]. The sender first uploads the model to the IPFS networks and obtains the IPFS hash as file identifier and the proof of storage. After that, the sender submits this IPFS hash to the smart contract. Later, the receiving party can query the IPFS hash from the smart contract and then download the model from the IPFS network using the obtained hash.

#### 3.3.2. How to Prevent Model Leakage?

During model distribution, attackers may steal the models by eavesdropping the communication channel between FL participants. Therefore, an encryption technique can be equipped in the FL system to protect the shared models.

**Applying Model Encryption**: Our survey shows three types of encryption researchers often use: public-key, symmetric-key, and homomorphic encryption.

*Public-Key Encryption*: Participants must share their public keys in the smart contract during registration. Anyone can then use those keys to encrypt the models before sending the models to the corresponding recipients [30].

*Symmetric-Key Encryption*: The public-key encryption can be considered heavy operations; therefore, if used frequently, it can create a bottleneck in the system. Following a well-known cryptography technique of sharing a secret [15], researchers can mix the public key with symmetric-key encryption to offload some heavy operations. Specifically, organizers must create a random secret key per task or global epoch. They then deliver the key to all trainers by encrypting it with the trainers’ public key. When sharing a model, organizers/trainers can use the shared secret key to encrypt and decrypt the model [36].

*Homomorphic Encryption*: Finally, homomorphic encryption (HE) can also be used as an alternative [41]. However, the primary purpose of using HE is to solve the private data leakage problem described in the subsequent section.

### 3.4. Training FL Models Strategy

Upon receiving global models, all eligible trainers train those models locally on their devices using their private data. Researchers can add several extra steps during this stage to solve several issues from the vanilla FL, described in the following paragraph.

#### 3.4.1. How to Prevent Data Leakage?

Carlini et al. [4] demonstrate that, even without access to the training data, attackers can still obtain the user-sensitive training data (e.g., credit card numbers) from the trained models through a membership inference attack. Our survey found two specific solutions to defend against this attack: differential privacy (DP) or HE.

**Using Differential Privacy**: Using DP, trainers add noises in the local model to confuse attackers when performing the inference attack on the trained model. The magnitude of the noise can be configured using a parameter known as *privacy budget*. If we increase this parameter value, our models become more private and robust against the membership attack. However, the models become less accurate when more noises are added [42].

**Using Homomorphic Encryption**: HE enables users to perform calculations on the cipher space without decrypting the data. With HE, the organizers first encrypt the global model with a HE-type private key and then distribute the encrypted models along with the HE-type public key to trainers. Trainers then perform direct training on the encrypted models by using the public key without decrypting them. This way, trainers cannot obtain the model parameters value, which makes them unable to perform the inference attack [37]. Unlike DP, HE does not modify the model parameters; hence, this approach has no training accuracy loss.

#### 3.4.2. How to Make Communication Efficient?

One of FL’s weaknesses is the number of communications needed to distribute the models among participants. As more clients join the system, more model exchanges will happen. Furthermore, the more complex the task is, the more model parameters will be generated, which results in a larger model size. For example, the AlexNet model size can reach 240 MB while the VGG-16 model size can reach 552 MB [43]. Therefore, communication costs become one of the possible bottlenecks in the system. To solve this issue, a model compression technique can be applied to the trained models before sharing them with others.

**Applying Model Compression**: BlockFLA [36] compresses the model binary parameters into base64 parameters. A model with 300 million parameters in binary representation can be formatted to 37.5 million bytes (or 75 million characters), achieving a 75% compression rate. Those bytes can then be compressed further into base64 characters, transforming the model into 50 million characters and achieving a total compression rate of 83.33%.

CREAT [44] directly integrates a novel compression technique into the model parameters. The authors first determine which parameters from the model are essential gradients. Those gradients must be kept as it is and reported precisely. On the other hand, the less essential gradients (those with values closer to 0) will be replaced with a default value. The authors use the K-Means clustering technique to cluster significant gradients and calculate centroid values. These centroid values will be reported in the system, which others can use to approximate the value of the true gradients from the same cluster. After compression, the time required to upload the models becomes significantly smaller [44].

### 3.5. Validating FL Models Strategy

Trainers may submit suboptimal models that are trained using poor datasets. They can also submit malicious models trained to corrupt the global model. Therefore, when aggregators receive trained local models from trainers, they can evaluate the models first before aggregating them into a new global model.

#### 3.5.1. Who Should Become Reviewers?

A third party must conduct the detection of malicious models as the peer-reviewer. We can see two design decisions depending on the total number of reviewers involved, including a single verifier or multiple reviewers.

**A Single Reviewer**: The first approach is to nominate a single node to be the reviewer. In this case, the aggregator is the most suitable candidate to become a reviewer since all trainers will eventually submit their local models to this entity [45].

**Multiple Reviewers**: A single reviewer raises a robustness issue. Therefore, researchers employ a contradictive strategy by allowing multiple participants to become reviewers.

*All Nodes Become Reviewers*: In this scenario, trainers must share their trained local models with other trainers for evaluation. Acting as reviewers, trainers then evaluate the shared models using their training data as a test dataset and share the evaluation scores with the smart contract. The smart contract then chooses suitable models to be aggregated into the global model through a contribution score algorithm [39].

*A Board of Reviewers*: While having the models peer-reviewed by all trainers increases the robustness of the evaluation process, it may not be efficient, especially when the FL system has many trainers. As an alternative, researchers employ a trade-off strategy by only selecting a few trainers as reviewers. Toyoda and Zhang [35] select eligible trainers randomly per epoch. The selected trainers in the next epoch will become reviewers of the current epoch and must evaluate the submitted models in this epoch. Meanwhile, [38,46] allow nodes to join the system as verifiers during the registration stage.

#### 3.5.2. How to Select Models for Aggregation?

After reviewers verify submitted models, they must choose which verified models are suitable for aggregation. The followings are several approaches used to determine model candidates.

**Random**: Kumar et al. [47] appoint the blockchain miner as the aggregator in their FL system. The chosen miner at each block selects *k* random trainers out of *N* total of trainers. Thus, only the trained models of the *k* selected trainers will be used for the aggregation. The authors use this randomization technique as a workaround to implement the differential privacy method. However, this method only prevents inference attacks on the global model but not the trained local models.

**Reporting**: BlockFLA [36] proposes a trojan detection algorithm and allows workers to verify each other submitted models. When a malicious trojan is detected on a particular model, reviewers can report this event as malicious behavior by invoking the penalty smart contract. The smart contract will settle this report and hold the trainer of that malicious model accountable if the report is valid. Models reported as malicious will not be used in the aggregation process.

**Voting**: Toyoda and Zhang [35] allow selected reviewers to vote for the most accurate models from all submitted local models. Each reviewer may only cast one vote for each model, and the system will choose the top-k most voted models as model candidates for aggregation.

**Contribution Scores**: The most common solution to select the trained models for aggregation is by using a pre-defined “rule” to calculate the contribution score for each submitted local model. Then, only models that pass the given threshold score will be chosen as model candidates. The following are a few examples of such contribution scores.

BlockFlow [39] performs a contribution evaluation by comparing medians from all of the submitted evaluation scores from reviewers. Based on those scores, the aggregator will most likely select models that generate higher accuracy. Similarly, SecCL [48] employs a Consensus Smart Contract (CSC) to solve a multi-winner problem based on model evaluation inputs from reviewers. Specifically, CSC calculates Broda positional scoring function (PSD) and then sorts all submitted evaluation scores to generate a top-k list of models with the highest accuracy. Based on this list, the aggregator can select only highly accurate models to update the global model. Learning markets [46] also allow reviewers to submit evaluation scores to the smart contract. The Moore-majority-vote and credit-scores-weighted-vote algorithms are then employed to get the consensus evaluation score from the submitted scores. After that, the smart contract evaluates how close the evaluation score for each model is to this consensus score. The models will not be selected in the aggregation if they are too far from the given threshold. Finally, Poster [37] and Mendis et al. [38] evaluate the models by comparing the performance of the global model before and after the aggregation trial. If the local models can improve the global model, they will be selected as model candidates.

#### 3.5.3. How to Punish Malicious Actors?

Once the submitted models are evaluated, researchers can punish participants who perform malicious behaviors. Two typical methods are commonly used in our surveyed papers: punish socially through a reputation system or economically by confiscating the deposited funds.

**Reputation System**: FL organizers can punish malicious participants by decreasing their reputation scores [46]. Recall that organizers can apply a minimum reputation score requirement during the registration to join the task. Therefore, reducing reputation scores can heavily impact participants, so they may be banned from the FL system. On the other hand, the system will increase participants’ reputation scores when no malicious behavior is detected.

**Deposit Mechanism**: Another alternative is to seize all deposited money when participants behave dishonestly [39]. When the system does not detect any malicious behavior from given participants, the deposited funds will be refunded fully to participants.

### 3.6. Aggregating FL Models Strategy

At the end of the FL stages, trainers or/and reviewers must deliver the trained models to the aggregator for aggregation.

#### 3.6.1. Who Can Become Aggregators?

Based on our survey, two variants exist on who should become aggregators.

**A Single Aggregator**: Vanilla FL [1] appoints a single centralized server as an aggregation server. Similarly, the same single-entity approach can be employed in the blockchain-based FL system [31].

The aggregator can also be chosen dynamically at each global epoch. Toyoda et al. [35] select the aggregator dynamically using a voting mechanism at each round. Participants must first vote for the top-k most accurate models for the given epoch. The system randomly chooses one of the model trainers among those selected models to become the aggregator. On the other hand, GFL [49] proposes to select an aggregator dynamically by appointing trainers with the most datasets at the given epoch to become the aggregator. Meanwhile, Kumar et al. [47] use the currently-selected blockchain miner to collect local updates and become the aggregators. Recall that the blockchain elects miners dynamically at each block. Therefore, the aggregator role will move from one node to another as the role of miners change at each block.

**Multiple Aggregators**: Because a single aggregator has the SPOF issue, researchers suggest using multiple aggregators to aggregate the global model [50]. Failures on one aggregator node now have little impact on the aggregation process. For this scenario to work, the aggregators must perform aggregation simultaneously and produce the same global model at each global epoch. Otherwise, a race condition must be settled before processing the next epoch. Training on different global models is ineffective and will likely corrupt the next one. Therefore, the smart contract is crucial in governing the aggregation in a multi-aggregator scenario.

#### 3.6.2. How to Aggregate Models?

When local models are verified and the participants have reached a consensus on who should become the aggregator, the chosen aggregator can start aggregating the validated models.

**Synchronous**: In synchronous aggregation, the FL process is usually divided into several stages, with a timeout in each stage. FL participants must perform the task at the given stage quickly before the deadline. If they miss the deadline, they may get a penalty and cannot continue to the rest of the stages, resulting in not obtaining the reward at the end of the stage.

Before the aggregators can aggregate the models, they must wait until the FL process reaches the aggregation stage. In other words, they must wait for all trainers to finish their training (and evaluation, if any) before forming the global model. This restriction is mostly by design and follows the type of FL algorithm the organizers use. For example, Federated Averaging [1] algorithm is based on synchronous aggregation. Therefore, studies developed using the Federated Averaging algorithm also indirectly employ the synchronous aggregation approach in their system.

**Asynchronous**: Contrary to the former approach, asynchronous aggregation requires no stage mechanism. At any given time, the trainer can join the FL task, get the initial model, train the model, and then freely submit the model to the aggregator. The system treats training from each participant independently. Therefore, the aggregator can instantaneously aggregate the received model without waiting for other trainers to complete their training.

From our surveys, only two papers employ this strategy. In [38], the authors employ a Fusion technique to aggregate the model. The aggregator extracts the features from the submitted models and then combines them with the previously submitted ones to create a concatenated feature layer. A fully connected neural network layer is then added to fuse those features. On the other hand, Chain FL [51] proposes an Online Federated Averaging algorithm, which tweaks the previous Federated Averaging [1] to work in an asynchronous scenario. Specifically, Chain FL tracks the number of data points used for a given local-update step to weigh each participant models correctly.

#### 3.6.3. Where Does Aggregation Happen?

Before the aggregators finally merge the local models, the final consideration is to choose whether they want to perform the aggregation locally on their machines (off-chain) or the blockchain (on-chain).

**Off-Chain**: The off-chain approach is not different from how the aggregators perform aggregation in a vanilla FL system. After such aggregation completes, the aggregator usually uploads the results to the blockchain so that they can be audited by other participants [48].

**On-Chain**: This second approach is practical when multiple aggregators are present in the system. Those aggregators must merge the models independently and simultaneously, while the global model result must be the same. A malicious aggregator may create a different global model than the rest to gain an advantage or jeopardize the system. Stem from this cause of problems, researchers propose to perform aggregation in the blockchain through the smart contract [52]. Because the smart contract can guarantee open, deterministic, and tamper-free code executions, aggregators should receive the same aggregation result from the smart contract.

## 4. Analysis on Investigated Design Patterns

Previous research [12] has studied several challenges and open problems for FL systems regarding robustness, efficiency, privacy, and fairness. This section elaborates on the investigated design pattern implications concerning those issues by analyzing the pros and cons of each design.

*Rules*: We give a plus (+1) point when we find advantages/benefits from committing to a particular design choice. Otherwise, we give a minus (−1) point when we find disadvantages or flaws. The given number indicates its significance. This way, we can see which design solves a given category of FL issues better than the rest. Finally, the cross sign (✗) indicates that the discussed design does not impact the given FL issues. In other words, this sign indicates that the mentioned design is on par with the vanilla FL design. We summarize our pros and cons discussions in Table 2.

### 4.1. Fundamental Metrics

The followings are fundamental FL issues from [12], which we use as metrics to analyze a particular design choice.

*Robustness*: The robustness of FL systems depends heavily on their ability to withstand malicious attacks. Adversaries can perform data poisoning to disrupt the aggregated global models. They may attack the global model by providing low-quality local models or performing malicious updates to corrupt the global model. Alternatively, adversaries can attack the aggregator to compromise the produced global model or disrupt the FL process. Robust FL systems should be able to handle all of those possible attacks.

*Efficiency*: The efficiency of the FL systems involves training and communication efficiency. The former investigates if participants can train or evaluate models without unnecessary complex procedures. The latter analyzes whether all transmitted models are distributed without duplications or many back-and-forth transmissions that cause additional delays. Thus, efficient FL systems should produce maximum results by consuming fewer resources.

*Privacy*: The FL systems should protect participants’ privacy by preventing data and model leakage. Adversaries should also not be able to get essential details from the system. For example, we must protect models from eavesdropping attacks. We must also guard the global model sharing such that only the authorized entity can get the models. Finally, detailed information regarding the identity of the trainers, reviewers, and aggregators should also be protected.

*Fairness*: Since model training is performed collaboratively, the system should be fair to everyone involved. The training’s success depends on whether its participants can contribute honestly without ill intentions and produce the most accurate model. Therefore, a fair FL system should reward honest participants while punishing malicious ones.

### 4.2. Analysis of Registering FL Clients Strategy

*How to attract trainers?*: The absence of reward will encourage malicious actions to emerge in the system. A simple flat reward can help to regulate the clients’ behavior to some extent, yet, because all clients receive the same reward, clients who put more effort into the training may feel disadvantaged. They may feel they have utilized more resources but receive the same compensation as the lower-effort ones. Among all available designs, the contribution-based reward is the fairest method because it incentives clients reasonably based on their contributions. However, this approach is also the most complex due to the requirements to measure each contribution, resulting in the worst efficiency.

*How to choose trainers?*: The open-trainer design is considered the fairest approach because the system treats anyone indifferently by allowing them to join the system freely. However, this “no-regulation” approach also means attackers can easily camouflage as legitimate users. Limiting the system access using the restricted-trainer design can restrain the attackers’ access and improve system robustness. However, additional authentication steps are required to filter legitimate users from malicious ones. Such verification requires clients to present some proof of identity (e.g., in the form of signatures [36]) or proof of capability (e.g., in the form of training and communication resources [34]). We must be aware that this information is intentionally leaked to the system, and adversaries can use this information to pinpoint the FL participants’ real identities.

### 4.3. Analysis of Distributing FL Models Strategy

*How to distribute models?*: Sending the models through the open channel can be considered a fragile and easy-to-corrupt method because there is no way we can audit the distributed models. In contrast, if we share a model through blockchain, participants can audit it safely because of the strong “ hard-to-tamper” guarantee that the blockchain provides. However, storing models in a public blockchain may incur expensive transaction fees because the model size can be huge (usually hundreds of megabytes for large neural network models [43]). Alternatively, if we save the models in a distributed storage (such as IPFS) and store only the model hash in the blockchain, we only need to store 32 bytes of data. This method is cheaper and way more efficient than storing the actual models while preserving the robustness benefits of the blockchain. Finally, FL participants must understand that storing the models through the blockchain or distributed storage means that anyone accessing the same blockchain or storage network can also obtain the shared model. Hence, these approaches protect less against model leakage than the open-channel method.

*How to prevent model leakage?*: The vanilla FL is not protected against model leakage, which causes the system becomes less robust, less private, and more unfair. Encryption can help to solve this problem, but we must also consider the additional computation required to execute such encryption, especially when we apply HE in our system, which is still inefficient in the current form [53].

### 4.4. Analysis of Training FL Models Strategy

*How to prevent data leakage?*: Even though we do not expose the private data to any third party when training using FL, adversaries may still be able to extract private information from the shared trained models through a membership inference attack [4]. Employing DP and HE can render this attack useless, although both strategies still have several drawbacks. The DP noise addition can deteriorate the accuracy of the trained models depending on the “privacy budget” configuration. A higher budget value makes the model more resistant to the inference attack at the cost of lower model accuracy [42]. On the other hand, HE still has low throughput [53]. Moreover, current HE is best at performing simple arithmetic but struggles to solve complex algorithms. Therefore, researchers often work around solutions to make HE work in FL. For example, Mendis et al. [38] apply Integer-Vector HE (IVHE) [54], which can only process calculations in the integer form. Meanwhile, model parameters are stored in a floating-point format. Therefore, additional conversion steps are needed to make IVHE work in their system.

*How to make communication efficient?*: We learned previously that the size of large neural network models could reach up to hundreds of megabytes [43]. When this statistic is combined with the requirement of FL that mandates models to be passed to one another frequently, the communication price can be costly, especially if the number of sharing is many (e.g., when we employ multiple trainers, reviewers, or aggregators). Model compression technique effectively reduces the communication overhead [36].

### 4.5. Analysis of Validating FL Models Strategy

*Who should become reviewers?*: The vanilla FL does not validate the submitted local models before aggregation, which makes it susceptible to data poisoning attacks through attacker submissions of malicious models. Applying a single reviewer to validate the trained models is the simplest and most effective solution. However, it may cause some fairness issues if that reviewer becomes malicious. The impact of the fairness issue decreases as we increase the number of available reviewers since now it is more difficult for adversaries to compromise multiple nodes. Finally, we must be aware that by allowing the models to be verified by reviewers, the trainers must agree and understand that the reviewers will obtain secrets about the trained models. This issue might become problematic if the system wants the trained local models to be private.

*How to select models for aggregation?*: All model selection algorithms used during verification can improve the system’s robustness. However, we notice that there is a trade-off between robustness and efficiency. Generally, more inputs are considered, makes the system more robust, but generates more processing delays. A random algorithm [47] only requires input from a single trustable random oracle. The reporting algorithm [36] must consider values between two entities, the accuser and the victim. Meanwhile, voting [35] and contribution score algorithm (e.g., in [39,46,48]) need to process inputs from all clients. Though including the same number of client inputs, the contribution score approach has a more complex algorithm than voting. Therefore, it can produce the highest robustness but requires the most resource among the alternatives.

*How to punish malicious actors?*: The vanilla FL does not employ any punishment strategy when they find malicious actors in the system. Adversaries can then abuse the system by not being willing to train honestly, significantly decreasing the system’s robustness and fairness. Employing a reputation and/or deposit mechanism can solve this issue. However, we must know that clients are most likely to use the same accounts every time they join the FL task when using a reputation system. Adversaries can potentially see the activity history of the clients because all information is recorded openly in the blockchain. In some FL cases, such information leaks may be undesirable.

### 4.6. Analysis of Aggregating FL Models Strategy

*Who can become aggregators?*: A single aggregator can be disruptive if adversaries can compromise the aggregator. Therefore, this approach has low robustness and fairness scores. On the other hand, multiple aggregators can solve them but result in efficiency and privacy consequences. Delivering models to many nodes may take time, especially if we have multiple trainers and aggregators (in order of hundreds or thousands). Moreover, trained local/global models will be shared with many nodes during the processes, potentially leaking valuable data.

*How to aggregate models?*: The synchronous or asynchronous aggregation does not directly impact blockchain usage because they are parts of the FL core design. However, committing to an asynchronous approach may result in a more efficient FL system since the aggregation can be done at any time in an atomic way.

*Where does aggregation happen?*: Applying aggregation off-chain is no different from how we aggregate models in the vanilla FL; therefore, it does not impact our evaluation metrics. On the other hand, system robustness is boosted when using an on-chain aggregation approach because it is tough to disrupt code executions in the blockchain. The system fairness is also augmented due to the easy-to-audit nature of blockchain. However, the current smart contract code operations are still very limited. For example, Machine learning models will most likely use floating points and arrays frequently. On the other hand, Ethereum does not fully support floating-point format [55], and array usages can be costly [56]. Because of this, the on-chain aggregation process can be less efficient than the off-chain approach. Moreover, the aggregation rules are known and open to all blockchain nodes. This public access may be unnecessary in some private FL scenarios.

### 4.7. Lesson Learned

We find several interesting insights from our pros and cons analysis of blockchain-based FL design patterns, which can be taken as a "lesson learned" to design future systems.

First, if we are looking for a robust FL model, we can also consider its fairness. In Table 2, among all positive (+) scores in fairness, 91% of them also have positive (+) scores in robustness. This relationship is expected because when we design a fair system, we also want to limit adversaries’ chance of gaining advantages in our system, which, in return, also boosts the overall robustness.

Second, we must be aware of privacy consequences when we aim for a robust FL model. Among all positive (+) scores in robustness from Table 2, 53% of them have negative (−) scores in privacy. Those problems stem from two major causes. First, the blockchain storage and smart contract execution are open to all blockchain nodes. Therefore, all design that relies on the "hard-to-tamper" guarantee of the blockchain to improve FL robustness must allow their model or algorithm to be exposed to all nodes. Second, the review process of trained local models mainly involves other nodes as third-party reviewers. Those reviewers must have access to the entire contents of the trained model to validate the models accurately.

Third, from Table 2, we can also see that among all positive (+) scores in robustness, 100% of them have negative (−) scores in efficiency. Likewise, from all positive (+) scores in privacy, 100% of them have negative (−) scores in efficiency. Furthermore, among all positive (+) scores in fairness, 91% of them have negative (−) scores in efficiency. This information highlights a trade-off between robustness, privacy, fairness, and efficiency. The more robust, private, and fair the system becomes, the more processes are needed, resulting in lower efficiency. System developers must be aware of this trade-off when designing their system, as pursuing a fair, private, and robust system altogether may not be ideal.

Fourth, from all previously surveyed papers, we only find two designs that improve system efficiency, which are a "model compression" design (c.f., [36,44]) and asynchronous aggregation. This statistic indicates that more efforts need to be made for future research in this area.

In conclusion, the most robust FL system is achievable through the combinations of contribution-based rewards, restricted-trainer, blockchain storage, data encryption, peer-reviewed model verification, deposit, reputation system, and multiple on-chain aggregators design. The most efficient FL system relies on model compression and asynchronous aggregation design. The most privacy-preserving FL system should include homomorphic encryption or differential privacy. Finally, for the fairest FL system, we can use the same features as in the most robust FL but replace the restricted-trainer with an open-trainer design.

## 5. Discussion

After introducing the design patterns of blockchain-based FL and analyzing their pros and cons, this section classifies the surveyed papers to find trends of design and implementation that are popular among the papers. This section also discusses the future research directions for blockchain-based FL research and mentions the limitations of our survey.

### 5.1. Classifications of Research Papers

We perform two classifications from surveyed papers regarding their designs and implementations.

#### 5.1.1. Classification of Design-Pattern

We read the surveyed papers and classified them based on found design patterns in Section 3. The summary of our investigations can be seen in Table 3. When a particular research paper applies a given design, we give the corresponding cell a checkmark (✓). Meanwhile, the cross (✗) indicates otherwise. From this classification, we can identify which designs gain lots of traction and which ones lack adoptions to be solved as future research directions.

*How to attract trainers?*: Among all papers with a reward system, the majority (66.67%) implement contribution-based rewards, with the most frequently-used method being dataset (37.5%) and accuracy measurement (also 37.5%). With the importance of incentives to motivate trainers, we think a reward system is a must-have feature, especially for blockchain-based FL, where the incentive mechanism can be built quickly by leveraging blockchain tokenization.

*How to choose trainers?*: The open-trainer design is favorable among surveyed papers (about 56.67%), while the rest uses the restricted-trainer method (43.33%). The deposit mechanism takes the lead (41.18%) in the latter design since, similar to incentive, the deposit can be easily implemented with blockchain tokenization. On the other hand, building a reputation system is the most complex approach; therefore, few papers (only 17.65%) employ this strategy despite its vast benefits.

*How to prevent model leakage?*: Only about half (53.33%) of our surveyed papers utilize encryption algorithms to protect the secrecy of the shared models in their system. Most use a public-key mechanism (about 45.83%), with HE implemented by 29.17%.

*How to distribute models?*: Because all the surveyed papers are blockchain-based systems, most (about 90.32%) leverage blockchain as a medium for model transfer, specifically, 48.39% of papers save the model directly in the blockchain, while 41.94% use IPFS as model storage and save the corresponding model hash in the blockchain.

*How to prevent data leakage?*: Among all the surveyed papers, only 36.67% apply DP or HE to prevent membership inference attacks. The membership attacks and their defenses are complex [67], and many papers are unwilling to perform those tasks. Moreover, this attack becomes less of a threat when running the FL system in a more trusted environment, such as in the cross-silo setting.

*How to make communication efficient?*: Model compression has not been used widely in surveyed papers; only about 6.67% of the papers implement such a compression scheme. Thus, this info can be seen as an opportunity window for future FL systems to focus more on the efficiency of the FL system.

*Who should become reviewers?*: Not many papers apply a verification mechanism to the trained local models before the aggregation (only about 53.33%). This may indicate that most papers are vulnerable to data or model poisoning attacks. Nevertheless, when deploying a future FL system, it is necessary to include model verification because it can still be effective as a safeguard to protect the quality of the global model, even when run in a trusted environment.

*How to select models for aggregation?*: The contribution score calculation over the trained models is used in most papers (about 60%) that employ the model verification scheme. Furthermore, most papers that apply model verification reward the clients based on their contribution with a contribution-based reward design.

*How to punish malicious actors?*: Unfortunately, most surveyed papers (66.67%) have no punishment for malicious behavior. Among all papers with punishment, deposit confiscation is used 66.67%

*Who can become aggregators?*: Researchers often choose a single aggregator (56.67%) over multiple aggregators (43.33%). A possible reason for this is that a single aggregator is closely similar to the vanilla FL. Hence, it is easier to implement than multiple ones.

*How to aggregate models?*: Most of the surveyed papers apply synchronous aggregation (93.33%), an aggregation process similar to vanilla FL. Therefore, future research is needed to apply asynchronous FL (as an alternative) for the blockchain-based system.

*Where does aggregation happen?*: Because the off-chain aggregation approach is straightforward and not different from the vanilla FL, many of the surveyed papers (about 66.67%) take this approach.

#### 5.1.2. Classification of Implementations

We analyze the trends of implementation procedures that the surveyed papers take in their experiment and derive possible research directions from this analysis. Our findings are summarized in Table 4. The checkmark and the cross sign have the same meaning as in Table 3.

Most surveyed papers (about 86.67%) have provided prototype implementation and evaluations. Based on their results alone, we can safely note that blockchain-based FL is highly viable.

*FL algorithms*: The vanilla Federated Averaging (FedAvg) algorithm [1] is used by most papers (69%). Aside from that, other FL algorithm is also used, such as Fusion/Ensemble, Online FedAvg [51], Model distillation [73], DANE [74], CDW FedAvg [33], Model chunking [52], and SignSGD [75]. This statistic also indicates that FL and blockchain are loosely coupled and can be set up plug-and-play.

*FL nodes*: Regarding the number of FL nodes used during the experiments, about 57.69% of papers employ up to 10 nodes, while 34.62% of papers run their experiments using 10 to 50 nodes, and 15.38% evaluate using 50 to 100 nodes. Meanwhile, only 7.69% leverage more than 100 nodes. Hence, future blockchain-based FL research should focus on using more nodes (in order of hundreds or even thousands) better to evaluate the feasibility and scalability of the system. Especially considering that a cross-device FL environment is expected to have an enormous number of nodes involved [23].

*FL library*: Most papers (about 26.92%) use Tensorflow, while 11.54% use PyTorch or Keras. Syft (7.69%) is also worth looking at in the future since this library focuses heavily on the FL environment. This library diversity indicates the versatility of the blockchain-based FL system.

*FL datasets*: Based on our observations, most papers evaluate their system for the image classification tasks using MNIST (46.15%) and CIFAR-10 (23.08%) datasets. Meanwhile, the rest of the papers evaluate diverse tasks such as health-related (in [31,39,60]) and also corporate-related ones (e.g., taxi [52] and air-conditioning [33] optimization). Future research should also explore many more areas that the surveyed papers in this study have not covered.

*Blockchain networks*: Most experiments are conducted locally, while only 11.54% use the Ethereum testnet. Even though there is no difference in the smart contract implementation for both networks, experimenting on the testnet will generate more delays than on the local. This delay may impact the scalability of the system. More evaluations on the testnet or even public networks are needed to assess the feasibility of the actual use case scenarios.

*Evaluation methods*: The surveyed papers commonly evaluate their system based on three categories: robustness (by evaluating the training accuracy), scalability (in terms of latency or throughput), and efficiency (by calculating the gas cost of methods in the smart contract). 88.46% of papers have widely evaluated the accuracy, proving that the accuracy of blockchain-based FL is on par with the vanilla FL. Meanwhile, only 50% of papers have scalability evaluation, and only 38.46% mention their gas cost measurements. Based on this statistic, future blockchain-based FL research should stop measuring the model accuracy unless they modify the FL-related parts in their system. Instead, more research is required to boost the system throughput and minimize the latency and gas cost.

*Availability*: Only 19.23% of papers open their implementation in the public repository. We think that more blockchain-based FL research should open their codes to the public to help future research better.

### 5.2. Ideal Design Choices

This part elaborates on the ideal architecture for future blockchain-based FL systems and strategic choices on which design to use.

First, the incentive is a mandatory feature to encourage honest behavior from participants. Especially the *contribution-based reward* design, which fairly rewards participants based on their performance. Similarly, *deposit* and/or *reputation system* should also be employed to protect against Sybil attacks.

Second, when using *homomorphic encryption*, we do not need to add *differential privacy* and other encryption schemes. Employing HE also does not reduce the trained global models’ accuracy as opposed to using DP. Therefore, HE should be the goal for future FL systems because it can solve security and privacy issues in one go.

Third, the *asynchronous aggregation* should be preferable over the synchronous ones. It provides participants flexibility regarding when they should download, train, review, and aggregate models. The process feels more seamless without time-gated operations as in the synchronous approach.

Fourth, the value of *model compression* increases if we use many trainers and reviewers in the system because those designs generate many back-and-forth model transfers between clients.

Finally, if using a public blockchain network, we want to limit on-chain operations such that the use of *open channel*, *third-party distributed storage (e.g., IPFS)*, *off-chain aggregation* becomes more appealing than the alternatives. Otherwise, we can process most things on-chain if using a private network for a better integrity guarantee.

### 5.3. Other Research Directions

FL and blockchain need a separate research direction on their own. First, FL research needs to produce more accurate, communication-efficient, and bias-tolerant algorithms [12]. Based on our survey results, the use of the asynchronous aggregation approach is still limited. Further aggregation schemes such as [51] can be explored more. On the other hand, blockchain research must continue to solve its scalability problem [76]. The layer-2 solutions, such as rollups [77], can be used to scale the blockchain network further. For example, Alief et al. [78] demonstrate that layer-2 blockchain with FL can help minimize the communication delay between clients and aggregators. By solving those FL and blockchain issues independently, we think the future blockchain-based FL system can also enjoy the benefits because blockchain and FL are loosely coupled.

The cross-silo and cross-device FL systems have their trade-offs. Applying cross-silo makes the FL system more efficient, but the training is more likely to be centralized. Meanwhile, the cross-device solution produces much overhead, but the training can be more decentralized. As demonstrated in [79], cross-silo and cross-device can be combined. The authors propose to separate the local model into two parts. The CNN feature extraction layers are trained in the local devices, while its fully-connected layers are trained in an edge server. Gaussian noise is then employed between those layers to prevent the edge server from stealing private data. This hybrid approach can offload heavy training requirements from devices to the server privately and securely. However, more investigation is required regarding its effectiveness and whether this split affects the accuracy in large models. Furthermore, more research is needed on whether this model splitting is feasible in other models besides the convolution neural network.

Finally, the ideal goal for a fair FL system has been described in [45], where (i) only FL organizers can obtain the trained global model, (ii) only the trainers can see the training data, and (iii) trainers cannot see each other trained local models. This requirement can only be solved by using HE. Even though the current implementation of HE is very inefficient in terms of throughput, we need to pay close attention to its progress in the future. We think improving HE can significantly enhance the quality of the blockchain-based FL system.

### 5.4. Limitations

Because this study only considers the Ethereum blockchain as one of the criteria for gathering papers to be surveyed, some non-Ethereum papers with exciting designs may not be included. Those papers usually employ outside-the-box architecture (e.g., custom transaction and block data structure, novel consensus), making it difficult to classify as no standardized blockchain architecture is available. Similarly, other blockchain platforms that support compatibility with Ethereum Virtual Machine (EVM), such as Polygon, Avalanche, and Klaytn, might also be overlooked. However, since they support EVM, design patterns from this paper can be applied to their networks out of the box when needed.

## 6. Conclusions

This paper performed a literature survey on many blockchain-based federated learning systems. First, we drew out about 31 design patterns from each research study in various FL workflows, such as registering clients, distributing models, training models, validating models, and the aggregation process. Second, we analyzed the pros and cons of committing to a particular design concerning its robustness, efficiency, privacy, and fairness. From our analysis, we found a linear relation between robustness and fairness in which, when we focused on improving the fairness of the FL system, we would end up with a robust system. We also discovered that designs that make the system more robust, private, and fair would suffer inefficiency due to the additional processing required. Therefore, pursuing excellent values on all metrics might not be feasible due to such trade-offs. Third, we classified the surveyed papers to find which designs were popular among surveyed papers. We found that more efforts must be made to improve system efficiency since only two designs (i.e., model compression and asynchronous aggregation) improve it, and only four papers adopt those designs. Finally, we investigated the surveyed papers based on their proof-of-concept implementations. The result shows diverse choices of FL algorithms, libraries, and datasets used in their experiments. Therefore, we can conclude that blockchain and FL are loosely coupled and can be used in various use cases (e.g., image classification, disease classification, and industrial optimization).

## Figures and Tables

**Table 1:** A summary of privacy (P), security (S), and trust (T) issues found in the vanilla federated learning system. Previous research has developed possible countermeasures, while the blockchain can be seen as a complementary tool to enhance those solutions.

Issues	Type	Possible Solutions	Blockchain Contributions
Model Leakage	P	Use encryption, client registration, and model auditing log.	Use the smart contract for registration and auditing.
Data Leakage	P	Employ differential privacy on local models.	-
Poisoning	S	Use reputation system, evaluate the local model updates before aggregation.	Build a reputation system and aggregation protocol on top of the smart contract.
Malicious Server	S	Make FL becomes less centralized.	Provide a platform for decentralization.
Low Motivations	T	Incentivize the clients based on their training contributions.	The digital token in the blockchain can be used as an incentive mechanism.
Client Dropouts	T	Employ deposit to punish intentional dropouts.	The digital token can also be used as a deposit mechanism.
Low Trust	T	-	Can be used as a trusted platform due to its high integrity guarantee.

**Table 2:** The pros (+) and cons (−) of committing to a particular design choice with respect to robustness (R), efficiency (E), privacy (P), and fairness (F). The given numbers indicate their significances.

Design	R	Reason	E	Reason	P	Reason	F	Reason
NoInc.	−1	encourage malicious behavior	✗	–	✗	–	−2	not fair for honest clients
Flat.	+1	encourage honest behaviour	−1	need little computation to distribute rewards	✗	–	−1	well-trained models subsidize poor-trained models
ConRe.	+2	encourage even honest behaviour	−2	need to calculate contributions from all clients	✗	–	+1	clients are rewarded based on their contributions
Open.	−1	most likely to include malicious trainers	✗	–	✗	–	+1	all clients can equally become trainers
Res.	+1	less likely to include malicious trainers	−1	must add filtering algorithm for trainer candidates	−1	clients need to disclose private info about themselves	−1	only qualified clients can become trainers
OC	−1	no log on models	✗	–	✗	–	−1	hard to audit
Blo.	+2	models are safely recorded in the blockchain	−2	storing huge models in the blockchain is costly	−1	all blockchain nodes can see the models	+1	all blockchain nodes can audit the models
IPFS	+1	hash of the model is stored in the blockchain	−1	storing only hashes is cheaper	−1	all blockchain and storage nodes can see the models	+1	all blockchain and storage nodes can audit the models
NoPrev.	−1	attackers can obtain models	✗	–	−1	attackers can obtain models	−1	attackers can obtain models
Enc.	+1	models are protected from third party	−1	additional steps are required for encryption	+1	models are protected from leakage	+1	only eligible entities can see the models
NoPrev.	✗	–	✗	–	−1	attackers may obtain private data	✗	–
DP	−1	decrease models’ accuracy	−1	additonal steps to add noise during training	+1	private data is secured	✗	–
HE	✗	does not affect robustness	−1	perform training on encrypted models is complex	+1	private data is secured	✗	–
NoComp.	✗	–	✗	–	✗	–	✗	–
Comp.	✗	–	+1	can safe a lot of bandwidth	✗	–	✗	–
NoVer.	−1	malicious models can jeopardize the global model	✗	–	✗	–	−2	malicious models may outperform honest models
Sin.	+1	models are verified by a reviewer	−1	require simple single-validation	−1	models are leaked to single reviewer	−1	may not fair if the reviewer is compromised
All.	+3	models are peer-reveiwed by all clients	−3	models must be transferred to all clients	−3	all clients know each others’ model	+2	hard to compromise when validated by all clients
Boa.	+2	models are verified by few reviewers	−2	models need to be delivered to few reviewers	−2	models are leaked to few reviewers	+1	sligthly difficult to compromise few reviewers
Ran.	+1	reducing the chance of malicious models to be selected	−1	need to have a trustable random oracle	✗	–	✗	–
Repo.	+1	reducing the chance of malicious models to be selected	−1	need to build a mediator for accusers and victims	✗	–	✗	–
Vot.	+1	reducing the chance of malicious models to be selected	−1	need to build a voting mechanism for all clients	✗	–	✗	–
Con.	+2	have a higher chance to exclude malicious models	−2	need to calculate contribution for each client	✗	–	✗	–
NoPun.	−1	may encourage malicous behaviour	✗	–	✗	–	−1	malicious entities does not get punishment
Repu.	+1	encourage honest behaviour	−1	needs additional credit score processing	−1	clients are most likely to use the same account	+1	malcious entity is punished socially
Depo.	+1	encourage honest behaviour	−1	needs additional deposit processing	✗	–	+1	malicious entity is punished economically
Sin.	−1	malicious aggregator can compromise the global model	✗	–	✗	–	−1	not fair if the aggregator is malicious
Mul.	+1	slightly difficult for a malicious aggregator to corrupt the global model	−1	models need to be distributed to all aggregators	−1	many nodes obtain information about the global models	+1	the robust aggregation process boosts clients’ trust
Syn.	✗	–	−1	must wait for slow trainers	✗	–	✗	–
Asy.	✗	–	+1	aggregate without waiting	✗	–	✗	–
Off.	✗	–	✗	–	✗	–	✗	–
On.	+1	the aggregation process becomes hard-to-tamper	−1	smart contract code execution is costly and complex	−1	all blockchain nodes can see the aggregation process	+1	the aggregation process can be audited

(1) *How to attract trainers?* **NoInc.** No incentive; **Flat.** Same reward; **ConRe.** Contribution-based reward; (2) *How to select trainers?* **Open.** Allow all; **Res.** Restricted trainer; (3) *How to distribute models?* **OC** Open channel; **Blo.** Blockchain; **IPFS** InterPlanetary File System; (4) *How to prevent model leakage?* **NoPrev.** No prevention; **Enc.** Use encryption; (5) *How prevent data leakage?* **NoPrev.** No prevention; **DP** Differential privacy; **HE** Homomorphic encryption; (6) *How to make communication efficient?* **NoComp.** No compression; **Comp.** Model compression; (7) *Who should become reviewers?* **NoVer.** No verfication; **Sin.** Single reviewer; **All.** All nodes are reviewers; **Boa.** A board of reviewers; (8) *How to select models for aggregation?* **Ran.** Random; **Repo.** Reporting; **Vot.** Voting; **Con.** Contribution scores; (9) *How to punish malicious actors?* **NoPun.** No punishment; **Dep.** Deposit mechanism; **Repu.** Reputation system; (10) *Who can become aggregators?* **Sin.** Single aggregator; **Mul.** Multiple aggregators; (11) *How to aggregate models?* **Syn.** Synchronous aggregation; **Asy.** Asynchronous aggregation; (12) *Where does aggregation happen?* **On.** On-chain; **Off.** Off-chain.

**Table 3:** A classification summary of our surveyed papers with respect to their design choices.

Ref.			Trainer Reward					Trainer Selection					Model Leak			Distribution		
			Flat	DQ	MQ	Vot.	Spe.	Open	Repu.	Dep.	Auth.	Lot.	Pub.	Sym.	HE	OC	Blo.	IPFS
[31]			✗	✓	✓	✗	✗	✗	✓	✓	✗	✗	✓	✗	✗	✗	✓	✗
[37]			✗	✗	✗	✗	✗	✓	✗	✗	✗	✗	✓	✗	✓	✗	✓	✓
[30]			✓	✗	✗	✗	✗	✓	✗	✗	✗	✗	✓	✓	✗	✗	✗	✓
[49]			✗	✗	✗	✗	✗	✓	✗	✗	✗	✗	✓	✗	✗	✗	✗	✓
[50]			✗	✗	✗	✗	✗	✓	✗	✗	✗	✗	✗	✗	✗	✗	✓	✗
[57]			✗	✗	✗	✗	✗	✓	✗	✗	✗	✗	✗	✗	✗	✗	✗	✓
[39]			✗	✓	✓	✗	✗	✗	✗	✓	✓	✗	✓	✗	✗	✗	✗	✓
[58]			✗	✗	✗	✗	✗	✗	✗	✗	✓	✗	✗	✗	✗	✗	✓	✗
[36]			✗	✗	✗	✗	✓	✗	✗	✗	✓	✗	✓	✓	✗	✗	✓	✗
[42]			✓	✗	✗	✗	✗	✓	✗	✗	✗	✗	✗	✗	✗	✗	✓	✗
[35]			✗	✗	✗	✓	✗	✗	✗	✗	✗	✓	✓	✓	✗	✗	✓	✗
[47]			✓	✗	✗	✗	✗	✗	✗	✓	✗	✗	✗	✗	✓	✗	✗	✓
[52]			✗	✗	✗	✗	✗	✓	✗	✗	✗	✗	✗	✗	✗	✗	✓	✗
[38]			✓	✗	✗	✗	✗	✗	✗	✓	✗	✗	✗	✗	✓	✗	✗	✓
[59]			✗	✗	✗	✗	✗	✓	✗	✗	✗	✗	✗	✗	✓	✗	✓	✗
[60]			✗	✗	✗	✗	✗	✓	✗	✗	✗	✗	✗	✗	✗	✗	✓	✗
[46]			✗	✗	✓	✗	✗	✗	✓	✓	✗	✗	✓	✓	✗	✗	✗	✓
[33]			✗	✓	✗	✗	✗	✗	✗	✗	✗	✓	✗	✗	✗	✓	✗	✗
[45]			✗	✓	✓	✗	✗	✓	✗	✗	✗	✗	✗	✗	✓	✗	✗	✓
[41]			✗	✗	✗	✗	✗	✓	✗	✗	✗	✗	✓	✓	✓	✗	✗	✓
[48]			✗	✗	✗	✗	✗	✓	✗	✗	✗	✗	✗	✗	✗	✓	✗	✗
[44]			✓	✗	✗	✗	✗	✓	✗	✗	✗	✗	✗	✗	✗	✗	✓	✗
[61]			✗	✗	✗	✗	✗	✓	✗	✗	✗	✗	✓	✓	✗	✗	✗	✓
[34]			✗	✓	✓	✗	✓	✗	✗	✗	✗	✓	✗	✗	✗	✓	✗	✗
[51]			✗	✗	✗	✗	✗	✓	✗	✗	✗	✗	✗	✗	✗	✗	✓	✗
[62]			✗	✗	✓	✗	✗	✗	✓	✗	✓	✗	✗	✗	✗	✗	✓	✗
[63]			✓	✗	✗	✗	✗	✓	✗	✗	✗	✗	✗	✗	✗	✗	✓	✗
[64]			✗	✗	✗	✗	✗	✓	✗	✗	✗	✗	✗	✗	✓	✗	✗	✓
[65]			✗	✗	✗	✓	✗	✗	✗	✓	✗	✗	✗	✗	✗	✗	✓	✗
[66]			✗	✓	✗	✗	✗	✗	✗	✓	✗	✗	✓	✗	✗	✗	✗	✓
Ref.	Data Leak		Effc.	Reviewer			Model Selection				Punishment		Aggregator		Method		Location	
	**DP**	**HE**	**Comp.**	**Sin.**	**All.**	**Boa.**	**Ran.**	**Repo.**	**Vot.**	**Con.**	**Dep.**	**Repu.**	**Sin.**	**Mul.**	**Syn.**	**Asy.**	**On.**	**Off.**
[31]	✗	✗	✗	✓	✗	✗	✗	✗	✗	✓	✓	✓	✓	✗	✓	✗	✗	✓
[37]	✗	✓	✗	✓	✗	✗	✗	✗	✗	✓	✗	✗	✓	✗	✓	✗	✗	✓
[30]	✗	✗	✗	✗	✗	✗	✗	✗	✗	✗	✗	✗	✓	✗	✓	✗	✗	✓
[49]	✗	✗	✗	✗	✗	✗	✗	✗	✗	✗	✗	✗	✓	✗	✓	✗	✗	✓
[50]	✗	✗	✗	✗	✗	✗	✗	✗	✗	✗	✗	✗	✗	✓	✓	✗	✓	✗
[57]	✗	✗	✗	✗	✗	✗	✗	✗	✗	✗	✗	✗	✗	✓	✓	✗	✗	✓
[39]	✓	✗	✗	✗	✓	✗	✗	✗	✗	✓	✓	✗	✗	✓	✓	✗	✗	✓
[58]	✗	✗	✗	✗	✗	✗	✗	✗	✗	✗	✗	✗	✗	✓	✓	✗	✓	✗
[36]	✗	✗	✓	✗	✓	✗	✗	✓	✗	✗	✓	✗	✗	✓	✓	✗	✓	✗
[42]	✓	✗	✗	✓	✗	✗	✗	✗	✗	✗	✗	✗	✓	✗	✓	✗	✓	✗
[35]	✗	✗	✗	✗	✗	✓	✗	✗	✓	✗	✗	✗	✓	✗	✓	✗	✗	✓
[47]	✓	✓	✗	✓	✗	✗	✓	✗	✗	✗	✓	✗	✓	✗	✓	✗	✗	✓
[52]	✗	✗	✗	✗	✗	✗	✗	✗	✗	✗	✗	✗	✗	✓	✓	✗	✓	✗
[38]	✗	✓	✗	✗	✗	✓	✗	✗	✗	✓	✓	✗	✓	✗	✗	✓	✗	✓
[59]	✗	✓	✗	✗	✗	✗	✗	✗	✗	✗	✗	✗	✗	✓	✓	✗	✗	✓
[60]	✗	✗	✗	✗	✗	✗	✗	✗	✗	✗	✗	✗	✗	✓	✓	✗	✓	✗
[46]	✗	✗	✗	✗	✗	✓	✗	✗	✗	✓	✓	✓	✓	✗	✓	✗	✗	✓
[33]	✗	✗	✗	✗	✗	✗	✗	✗	✗	✗	✗	✗	✓	✗	✓	✗	✗	✓
[45]	✗	✓	✗	✓	✗	✗	✗	✗	✗	✓	✗	✗	✗	✓	✓	✗	✓	✗
[41]	✗	✓	✗	✗	✗	✗	✗	✗	✗	✗	✗	✗	✓	✗	✓	✗	✗	✓
[48]	✗	✗	✗	✗	✓	✗	✗	✗	✗	✓	✗	✗	✓	✗	✓	✗	✗	✓
[44]	✗	✗	✓	✗	✗	✗	✗	✗	✗	✗	✗	✗	✓	✗	✓	✗	✗	✓
[61]	✓	✗	✗	✗	✗	✗	✗	✗	✗	✗	✗	✗	✓	✗	✓	✗	✗	✓
[34]	✗	✗	✗	✓	✗	✗	✗	✗	✗	✓	✗	✗	✓	✗	✓	✗	✗	✓
[51]	✗	✗	✗	✗	✗	✗	✗	✗	✗	✗	✗	✗	✗	✓	✗	✓	✓	✗
[62]	✓	✗	✗	✗	✓	✗	✗	✓	✗	✗	✗	✓	✗	✓	✓	✗	✓	✗
[63]	✗	✗	✗	✗	✓	✗	✗	✓	✗	✗	✗	✗	✗	✓	✓	✗	✓	✗
[64]	✗	✓	✗	✗	✗	✗	✗	✗	✗	✗	✗	✓	✓	✗	✓	✗	✗	✓
[65]	✗	✗	✗	✗	✗	✓	✗	✗	✓	✗	✓	✗	✗	✓	✓	✗	✗	✓
[66]	✗	✗	✗	✓	✗	✗	✗	✗	✗	✓	✓	✗	✓	✗	✓	✗	✗	✓

(1) *How to attract trainers?* **Flat** Same reward; **DQ** Dataset or **MQ** Model quality; **Vot.** Voting; **Spe.** Speed; (2) *How to select trainers?* **Open** Allow all; **Repu.** Reputation; **Dep.** Deposit; **Auth.** Authentication; **Lot.** Lottery; (3) *How to prevent model leakage?* **Pub.** Public-key, **Sym.** Symmetric-key, or **HE** Homomorphic encryption; (4) *How to distribute models?* **OC** Open channel; **Blo.** Blockchain; **IPFS** InterPlanetary File System; (5) *How prevent data leakage?* **DP** Differential privacy; **HE** Homomorphic encryption; (6) *How to make communication efficient?* **Comp.** Model compression; (7) *Who should become reviewers?* **Sin.** Single reviewer; **All.** All nodes are reviewers; **Boa.** A board of reviewers; (8) *How to select models for aggregation?* **Ran.** Random; **Repo.** Reporting; **Vot.** Voting; **Con.** Contribution scores; (9) *How to punish malicious actors?* **Dep.** Deposit mechanism; **Repu.** Reputation system; (10) *Who can become aggregators?* **Sin.** Single aggregator; **Mul.** Multiple aggregators; (11) *How to aggregate models?* **Syn.** Synchronous aggregation; **Asy.** Asynchronous aggregation; (12) *Where does aggregation happen?* **On.** On-chain; **Off.** Off-chain.

**Table 4:** A classification summary of our surveyed papers with respect to their implementation characteristic.

Ref.	Environment				Evaluation			Dependencies			Ava.
	Impl.	L	T	# FL Nodes	Acc.	Sca.	GC	FL Algorithm	ML Library	ML Dataset	
[31]	✓	✓	✗	3	✓	✓	✓	FedAvg	SYFT	BCWD, HDD	✗
[37]	✓	✓	✗	up to 40	✗	✓	✗	FedAvg	PyTorch	BCWD	✗
[30]	✗	✗	✗	✗	✗	✗	✗	✗	✗	✗	✗
[49]	✓	✓	✗	✗	✓	✗	✗	FedAvg, Distilation	✗	MNIST, CIFAR-10	[68]
[50]	✗	✗	✗	✗	✗	✗	✗	DANE	✗	✗	✗
[57]	✓	✗	✓	3	✓	✗	✓	FedAvg	Tensorflow	MNIST	[69]
[39]	✓	✗	✗	up to 100	✓	✗	✓	FedAvg	✗	Adult, KDD	✗
[58]	✓	✓	✗	10	✓	✗	✗	FedAvg	✗	✗	✗
[36]	✓	✓	✓	up to 10	✗	✓	✓	FedAvg, signSGD	✗	CIFAR-10	✗
[42]	✓	✗	✗	up to 300	✓	✗	✗	FedAvg	Tensorflow	MNIST, CIFAR-10	✗
[35]	✗	✗	✗	✗	✗	✗	✗	FedAvg	✗	✗	✗
[47]	✓	✗	✗	up to 5	✓	✗	✗	FedAvg	Hyperas	MNIST	✗
[52]	✓	✓	✗	up to 128	✓	✓	✓	Model chunking	Keras, Tensorflow	NYC Taxi	✗
[38]	✓	✓	✗	up to 6	✓	✓	✗	Fusion	Tensorflow	MNIST, NASA Glenn	✗
[59]	✓	✓	✗	10	✓	✓	✗	FedAvg	Tensorflow	PASCAL VOC 2012	✗
[60]	✓	✓	✗	15	✓	✗	✗	FedAvg	Numpy	NIDDK	[70]
[46]	✓	✓	✗	up to 7	✓	✗	✓	Fusion/Ensemble	Keras	MNIST	✗
[33]	✓	✓	✗	5	✓	✓	✗	CDW FedAvg	Leaf	Air Conditioning Data	✗
[45]	✗	✗	✗	✗	✗	✗	✗	✗	✗	✗	✗
[41]	✓	✓	✗	up to 11	✓	✗	✓	FedAvg	Numpy	MNIST	✗
[48]	✓	✓	✗	up to 30	✓	✗	✗	FedAvg	Keras, sklearn	MNIST	✗
[44]	✓	✗	✗	30	✓	✓	✗	FedAvg	✗	MovieLens	✗
[61]	✓	✓	✓	5	✓	✓	✓	FedAvg	✗	MNIST	✗
[34]	✓	✓	✗	✗	✗	✗	✗	✗	✗	✗	✗
[51]	✓	✓	✗	✗	✓	✗	✗	Online FedAvg	Tensorflow, PyTorch	MNIST, CIFAR-10	✗
[62]	✓	✓	✗	up to 30	✓	✓	✓	FedAvg	Tensorflow	MNIST, BelgiumTS	✗
[63]	✓	✓	✗	8	✓	✓	✗	Zokrates Learning	✗	UCI Daily Sport	[71]
[64]	✓	✓	✗	✗	✓	✓	✓	✗	✗	Diabetes, Cancer	✗
[65]	✓	✓	✗	50	✓	✓	✗	FedAvg	PyTorch, PySyft	MNIST	✗
[66]	✓	✓	✗	5	✓	✗	✗	✗	✗	CIFAR, TinyImageNet	[72]

**Impl.** Whether the prototype implementation is presented or not; **L** Local network; **T** Test network (testnet); **Acc.** Accuracy; **Sca.** Scalability; **GC** Gas Cost; **Ava.** Whether the source code for the project is available for the public or not.

## Data Availability

Not applicable.
